# GAC: Gene Associations with Clinical, a web based application

**DOI:** 10.12688/f1000research.11840.4

**Published:** 2018-02-15

**Authors:** Xinyan Zhang, Manali Rupji, Jeanne Kowalski

**Affiliations:** 1 Winship Cancer Institute of Emory University, Atlanta, GA, 30322, USA; 2 Department of Biostatistics and Bioinformatics, Rollins School of Public Health, Emory University, Atlanta, GA, 30322, USA

**Keywords:** SuperPC, binary outcome, continuous, time-to-event, forest plot

## Abstract

We present GAC, a shiny R based tool for interactive visualization of clinical associations based on high-dimensional data. The tool provides a web-based suite to perform supervised principal component analysis (SuperPC), an approach that uses both high-dimensional data, such as gene expression, combined with clinical data to infer clinical associations. We extended the approach to address binary outcomes, in addition to continuous and time-to-event data in our package, thereby increasing the use and flexibility of SuperPC.  Additionally, the tool provides an interactive visualization for summarizing results based on a forest plot for both binary and time-to-event data.  In summary, the GAC suite of tools provide a one stop shop for conducting statistical analysis to identify and visualize the association between a clinical outcome of interest and high-dimensional data types, such as genomic data. Our GAC package has been implemented in R and is available via http://shinygispa.winship.emory.edu/GAC/. The developmental repository is available at https://github.com/manalirupji/GAC.

## Introduction

Heterogeneity in terms of tumor characteristics, prognosis, and survival among cancer patients has remained a persistent problem. It has been well established that clinical factors alone are not sufficient to explain differences in prognosis. For example, based on clinical factors only, two tumor patients may have the same prognosis, but may not respond to the same treatment as the tumors may have a completely different molecular composition ^1– 5^. Despite the introduction of a tumor’s genomic profile to explain differences in prognosis, there remains unexplained heterogeneity in tumor response to treatment. One factor potentially attributing to such unexplained differences may be due to inaccurate prognosis and prediction resulting from the analysis approach used to define prognostic markers of response.

For this purpose, Bair and Tibshirani ^6^ introduced a supervised principal component (SuperPC) method within the context of defining expression-based cancer subtypes of prognostic significance. The method uses both gene expression and clinical data for predicting patient prognosis. This approach was applied to several publicly available datasets that demonstrated it’s ability to accurately predict the clinical outcome of interest based on a given gene expression profile. Since its inception, SuperPC has been introduced as a powerful tool for reducing dimensionality in selecting features (a.k.a., genes) of prognostic relevance in cancer.

Currently, the SuperPC method has been developed as an R package, ‘superpc’ but as it stands, it is unable to address the following:

1) clinical association based on a binary outcome (e.g. responders versus non-responders);
2) ease of use for clinicians and researchers with limited programming skills; and
3) a visual summary of results.

To address these limitations, we developed GAC: Gene Association with Clinical, an interactive, GUI-based web-based application for analysis of gene associations with various clinical outcomes of interest. We developed GAC based on the R packages ‘shiny’ and ‘superpc’. Our GAC tool enables the user to perform a SuperPC analysis for three types of outcomes: time-to-event, continuous, and binary, and provides a summary of results using forest plots that may be readily exported into a file.

## Methods

### Supervised principal component analysis

SuperPC is a generalization of principal component analysis, which generates a linear combination of the features or variables of interest that capture the directions of largest variation in a dataset. Instead of using the whole dataset directly, SuperPC defines a list of genes based on their association with an outcome of interest. To select the list of genes, a univariate score for each gene is calculated and those features (a.k.a., genes) whose score exceeds a threshold are retained as input into a principal component analysis, based on the retained features. For details, refer to Bair and Tibshirani ^6^.

### Time-to-event outcome

**Figure 1.**
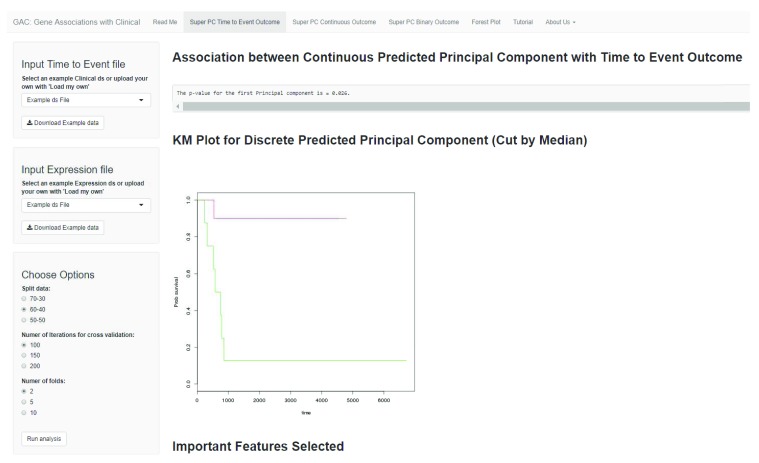
SuperPC time-to-event outcome. The interface shows an example SuperPC for time-to-event outcome. The left panel allows the user to select the various options such as data split, the optimal threshold, number of folds and a choice of generating new fold IDs or using a pre-existing set to replicate results. The right panel includes the results of the analysis. Use the ‘Run Analysis’ button (in left panel) to display results based on updated option(s). The KM plot displays the association of the outcome with the predicted principal component by the median. In addition, the univariate analysis regression scores and tables are also available for download.

SuperPC for time-to-event was conducted using the ‘superpc’ package in R. Depending on the sample size of the original dataset; the researcher selects what proportion of the dataset to split into training and testing. The researcher can also specify how many numbers to test to check which the optimal threshold is. The number of folds for cross validation to determine the threshold also needs to be determined. There is also an option to run the analysis randomly, or upload fold IDs to replicate an analysis that was previously carried out. The association between the time-to-event outcome and the predicted principal component may be represented in a KM plot by dichotomizing the principal component using the median ( Figure 1).

### Continuous outcome

**Figure 2.**
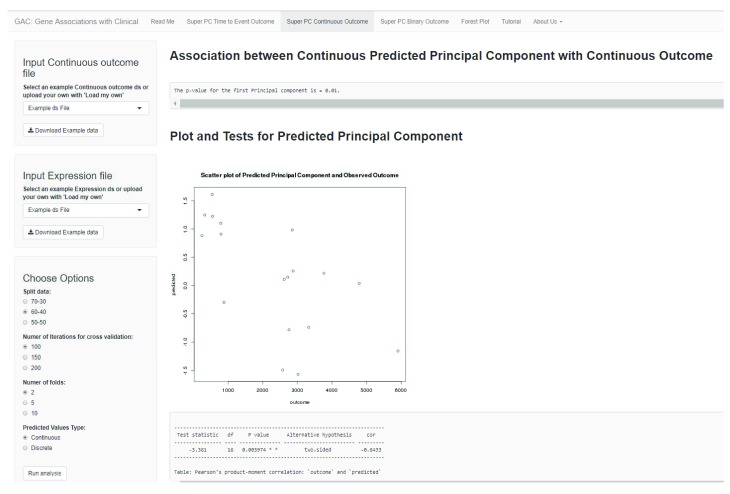
SuperPC continuous outcome. The interface shows an example SuperPC for continuous outcome. Similar to Figure 1, the user can choose the appropriate settings using the left panel and view the results of the analysis to the right. The association of the continuous outcome with the predicted principal component is summarized using a scatter plot (as seen). The user can alternatively choose to summarize these results through boxplots by dichotomizing the predicted principal component based on the median. As in Figure 1, the univariate regression scores and plots are available for download in the left panel.

SuperPC for continuous outcomes is implemented using the ‘ *superpc*’ package in R, with the same options as time-to-event analysis. The predicted principal component is presented visually as continuous values through a scatter plot along with Pearson’s correlation ( Figure 2). The predicted principal component could also be presented as binary groups (cutoff at median) through a boxplot, with a t-test applied.

### Binary outcome

**Figure 3.**
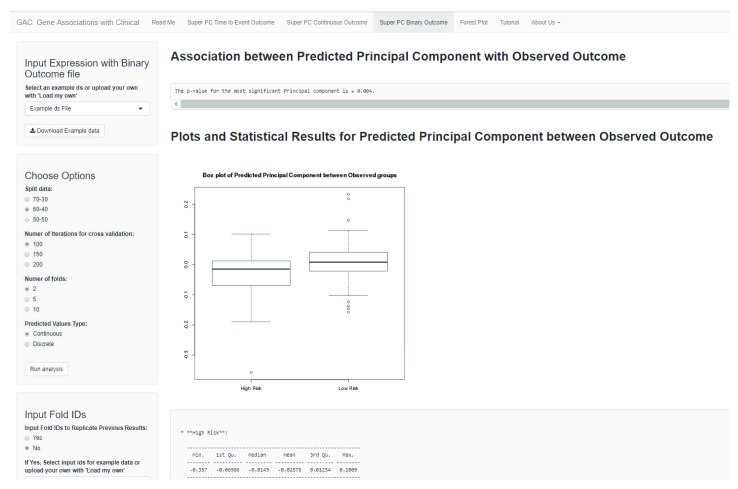
SuperPC binary outcome. The interface shows an example SuperPC for binary outcome. The user can opt for similar options as in the previous figures. Also, as in Figure 2, the user has a choice to display the continuous predicted principal component as a scatter plot, or divide it into binary discrete groups (using median cut-off) to represent the association through a barplot. Similar download options are available.

In the ‘superpc’ R package developed by Bair and Tibshirani (2004), SuperPC analysis can be performed on both continuous and survival outcomes. We have extended this tool to include SuperPC for binary outcome (example ‘responders’ vs ‘non-responders’). This extension follows a similar analysis workflow as the other two outcomes in that a list of genes is defined based on a univariate score to which a threshold is applied and the genes whose scores exceed the threshold are used as input into a principal component analysis. For modeling gene associations with binary outcomes a logistic regression has been implemented. The predicted principal component can be visualized as either a continuous variable through a box plot, with a t-test to summarize the statistical association ( Figure 3), or as binary groups (cutoff at median) using a bar plot, with a chi-square test to summarize the statistical association between the predicted and the observed outcome.

### Forest plot

**Figure 4.**
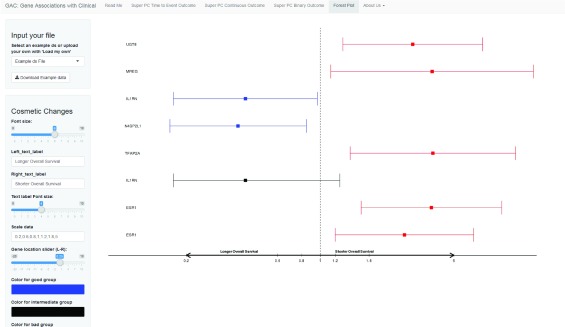
Forest plot. The interface shows an example forest plot. The left side comprises a user menu and the right includes the result plot. Users can upload their own summarized results with hazard ratio and confidence intervals for the survival outcome, or odds ratio and confidence intervals for the binary outcome. For graphical display, the researchers could choose to input different labels, font sizes and colors.

A forest plot is a graphical display of point estimates of association widely used in meta-analysis. It has become popular for displaying the associations between clinical and genomic data. With our GAC tool, users have the option to generate a forest plot to display results ( Figure 4).

### Implementation

The GAC tool is written in R and tested using version 3.3.0. The interactive plots and data tables are made available using the shiny R package ( www.rstudio.com/shiny).

### Operation

Using a windows 7 Enterprise SP1 PC with a 32.0 GB RAM and a 3.30 GHz Intel ^®^ Xeon ^®^ Processor E5 Family, the time-to-event and regression analysis with 45 patients from the TCGA BRCA dataset was completed in 2.54 s and 1.80 s, respectively. The binary outcome analysis using the CoMMPass data of 135 patients was completed in 65.05 s. Source code is available at https://doi.org/10.5281/zenodo.1064841.

## Discussion

GAC is a suite of tools that allows the user to conduct statistical analysis to identify and visualize the association between clinical outcomes of interest and genomic data using an interactive application in R.

## Data and software availability

For SuperPC time-to-event and regression analysis, we used TCGA BRCA RNASeqV2 gene expression and clinical data, downloaded from the TCGA data portal (now accessed at https://portal.gdc.cancer.gov/) ^7^. The data included 380 differentially expressed genes when favorable (patients who did not die with at least 7 years of follow up) and unfavorable (patients who died 30 months post-treatment) outcomes from 45 patients were compared.

For SuperPC binary outcome analysis, CoMMPass IA9 RNASeq expression and clinical data was downloaded from the publicly available Multiple Myeloma Research Foundation (MMRF) database ( https://research.themmrf.org/rp/download). Patient cytogenetics for outcome dichotomization was obtained from the MMRF data portal’s ‘Analysis Tools’ section ( https://research.themmrf.org/rp/explore/). 135 patients with clinical, gene expression and copy number data were classified as high risk based on cytogenetics and the remaining 343 as not high risk. Among these patients, the top 1450 most variable genes were used.

The developmental repository is available at https://github.com/manalirupji/GAC.

The example data used in this article is available in Zenodo. User uploaded data should be in the same format as the example data provided.

Archived source code as at the time of publication: https://doi.org/10.5281/zenodo.1064841 ^8^

License: GAC is available under the GNU public license (GPL-3).
