# Supplementary material for: GAC: Gene Associations with Clinical, a web based application
Source: F1000Res. 2018 Feb 15;6:1039. Originally published 2017 Jul 3. [Version 4] doi: 10.12688/f1000research.11840.4 (PMC5658710; doi:10.12688/f1000research.11840.4)
Supplement: Peer review history

# Peer review: Referee response for version 3

**DOI:** 10.5256/f1000research.14759.r29420

**Published:** 2018-01-08

**Authors:** Matthew N. McCall

**Affiliations:** 1 Department of Biostatistics and Computational Biology, University of Rochester Medical Center, Rochester, NY, USA

I thank the authors for incorporating my suggestions into their work; however, the figures in the paper should be updated to reflect the changes in the shiny app, which were made in response to comment 2 from my initial review:

 "2. The numeric output of the GAC shiny app is often unformatted R output. For example, on the Super PC Binary Outcome tab below the box plots. It would be better to provide formatted results (e.g. using knitr::kable). Also, the p-values (and other numeric outputs) should likely be rounded to a number of significant digits that conveys the precision of the values."

I have read this submission. I believe that I have an appropriate level of expertise to confirm that it is of an acceptable scientific standard.

# Response

**Published:** 2018-02-15

**Authors:** Manali Rupji

**Affiliations:** Emory University, USA

We thank the referee for their suggestions which have lead to an improved paper. In the latest version, we have incorporated updates to Figures 1-3 to reflect the changes to output formatting made in version 3.

# Peer review: Referee response for version 2

**DOI:** 10.5256/f1000research.13806.r26969

**Published:** 2017-10-26

**Authors:** Cedric Simillion

**Affiliations:** 1 Interfaculty Bioinformatics Unit and SIB Swiss Institute of Bioinformatics, University of Bern, Berne, Switzerland

I agree with the previous referee that the authors have developed a nice user interface to the SuperPC algorithm, which should make its application accessible to many experimental scientists. I read the manuscript and I have little to no comments to make on its contents.

 However, I have tried to use the tool myself on my own dataset and was unable to get it to work. I had formatted my data exactly as in supplied example input files, even with the exact same column names. After submitting my data, I only got an unhelpful error message "ERROR: An error has occurred. Check your logs or contact the app author for clarification." I could not see any logs or any other means of finding out why the program didn't work.

 If the problem lies with the formatting of the input files, the authors need to provide some more documentation on the format of the input data, including column names, newline convention, etc.

 Given that I presently cannot verify that the tool is working properly, I therefore cannot accept the manuscript for indexing.

I have read this submission. I believe that I have an appropriate level of expertise to confirm that it is of an acceptable scientific standard, however I have significant reservations, as outlined above.

# Response

**Published:** 2017-12-21

**Authors:** Manali Rupji

**Affiliations:** Emory University, USA

We thank the reviewer for their thoughtful comments. 

 Below is our response to the reviewer’s comments :

 Thank you for pointing this problem out.  As it turns out, there was indeed a bug with reading the input data that caused the problem described.  This bug has been fixed in the new version of the tool available on the same link http://shinygispa.winship.emory.edu/GAC/.

 Additionally, we have included a sentence about data formatting in the Data and software availability section: ‘User uploaded data should be in the same format as the example data provided.’ The tutorial has also updated to state the same.

 The tool has also been updated to indicate errors in code directly as seen on the R console as a way to better inform on the possible nature of the error.

# Peer review: Referee response for version 2

**DOI:** 10.5256/f1000research.13806.r26812

**Published:** 2017-10-23

**Authors:** Matthew N. McCall

**Affiliations:** 1 Department of Biostatistics and Computational Biology, University of Rochester Medical Center, Rochester, NY, USA

The authors describe a software package for interactive (via a shiny webapp) use of the superpc R package. This work will likely make the methods in the superpc package available to users who are unable to work in R. 

 My primary criticism of this work is that there does not seem to be a clear description of the methodology to which GAC provides GUI access. I understand that these methods were not developed by the authors of this paper; however, if the target audience are clinician scientists, it is doubtful that will have read the original paper ^1^.

 A lay description of the method would greatly improve this manuscript.

 I also have a few minor criticisms of the article:

1. The authors write, “Currently, prognosis and outcome predictions are solely based on clinical factors.” This seems to dismiss a lot of recent work in this area. On a related note, the most recent paper cited is from 2004.  
2. The numeric output of the GAC shiny app is often unformatted R output. For example, on the Super PC Binary Outcome tab below the box plots. It would be better to provide formatted results (e.g. using knitr::kable). Also, the p-values (and other numeric outputs) should likely be rounded to a number of significant digits that conveys the precision of the values.
3. There are several grammatical errors. For example:
  “The researcher can also specify how many numbers to test to check which the optimal threshold is.”
4. The superPC method is referred to in a variety of ways; it would be good to pick one of these: 
  “Our GAC tool enables the user to perform a SuperPC analysis...”
  “... super pc analysis can be performed on both continuous …” “We have extended this tool to include super PC…” 
  “Figure 2. SuperPC continuous outcome”

I have read this submission. I believe that I have an appropriate level of expertise to confirm that it is of an acceptable scientific standard, however I have significant reservations, as outlined above.

## References

[1] BairETibshiraniR: Semi-supervised methods to predict patient survival from gene expression data. *PLoS Biol*.2004;2(4) : 10.1371/journal.pbio.0020108 E108 10.1371/journal.pbio.0020108 15094809PMC387275

# Response

**Published:** 2017-12-21

**Authors:** Manali Rupji

**Affiliations:** Emory University, USA

We thank the reviewer for their thoughtful comments that have led to both an improved paper and tool. 

 We have included in the introduction section a brief summary of the 'superpc' method. 

 Additionally, below, we respond individually to each comment.

 1. The authors write, “Currently, prognosis and outcome predictions are solely based on clinical factors.” This seems to dismiss a lot of recent work in this area. On a related note, the most recent paper cited is from 2004. 

 We agree with the reviewer and have updated the introduction.Considering that the main method, supervised principal components analysis, was published in 2004, the other papers referenced earlier reflect the cited results of the method.

 2. The numeric output of the GAC shiny app is often unformatted R output. For example, on the Super PC Binary Outcome tab below the box plots. It would be better to provide formatted results (e.g. using knitr::kable). Also, the p-values (and other numeric outputs) should likely be rounded to a number of significant digits that conveys the precision of the values. 

 We have modified the output in both the SuperPC binary and continuous outcome sections to provide formatted results using pander from the ‘knitr’ package. which also provides greater control on the display of results. The numeric output has also been modified as suggested by provided rounded results in the table.

 3. There are several grammatical errors. For example: “The researcher can also specify how many numbers to test to check which the optimal threshold is.”

 We have corrected the grammatical errors in the manuscript.

 4. The superPC method is referred to in a variety of ways; it would be good to pick one of these:“Our GAC tool enables the user to perform a SuperPC analysis...”

 “... super pc analysis can be performed on both continuous …” “We have extended this tool to include super PC…”

 “Figure 2. SuperPC continuous outcome”

 We thank the reviewer for pointing out this inconsistent nomenclature.  When specifically referring to the R package, we use the name of the package as ‘superpc’ and when referring to the tool, method or software, ‘SuperPC’ is used throughout the manuscript.

# Peer review: Referee response for version 2

**DOI:** 10.5256/f1000research.13806.r26394

**Published:** 2017-09-27

**Authors:** Shengjie Yang

**Affiliations:** 1 Program for Computational Genomics & Medicine, NorthShore University HealthSystem, Evanston, IL, USA

The authors have addressed my concerns appropriately in the revised version and this work is worthy of indexing.

I have read this submission. I believe that I have an appropriate level of expertise to confirm that it is of an acceptable scientific standard.

# Response

**Published:** 2017-09-29

**Authors:** Manali Rupji

**Affiliations:** Emory University, USA

We thank the referee for taking the time to review our work, and thank them for the kind review.

# Peer review: Referee response for version 1

**DOI:** 10.5256/f1000research.12794.r26010

**Published:** 2017-09-18

**Authors:** Shengjie Yang

**Affiliations:** 1 Program for Computational Genomics & Medicine, NorthShore University HealthSystem, Evanston, IL, USA

The authors did a nice job to develop a web tool called GAC, which is used to explore the association between clinical and genomic data. Essentially, GAC is a GUI of R package ‘superpc’. They not only provided a handy web tool but also published the source code to allow researchers to do some further expansion according to their own specific study objects. The manuscript is well written, but I have some comments:

1. Since the example data is from TCGA and this work aims to facilitate clinicians and researchers with limited programming skills, it is necessary to cite some literature to tell readers how to retrieve and process the TCGA Data, such as TCGA-Assembler (Nature methods, 2014), and it would be better if such tools can be embedded into GAC.
2. The authors stated that ‘superpc' is unable to address the limitation about clinical association based on a binary outcome in the Introduction. But in the Methods section, it said ‘SuperPC for binary outcome follows the same analysis workflow as the other two outcomes’. It confuses me and please explain the details.
3. I find that the calculation will be automatically triggered when changes any one option in the sidebar. That is not an user-friendly experience, especially it happens in the ’Super PC Binary Outcome’ module which can not return the result immediately. Think about that if the users want to change two default options, they have to adjust the first one, wait a few seconds and then adjust the second option, which is not efficient. So adding a ‘submit’ button to trigger the calculation is a better way.
4. Build a user manual page to explain the details of each function and use an example to demonstrate the usage.

I have read this submission. I believe that I have an appropriate level of expertise to confirm that it is of an acceptable scientific standard, however I have significant reservations, as outlined above.

# Response

**Published:** 2017-09-20

**Authors:** Manali Rupji

**Affiliations:** Emory University, USA

We thank the reviewer for their thoughtful comments that have led to an improved paper.  Below, we respond individually to each comment.

 

1. Since the example data is from TCGA and this work aims to facilitate clinicians and researchers with limited programming skills, it is necessary to cite some literature to tell readers how to retrieve and process the TCGA Data, such as TCGA-Assembler (Nature methods, 2014), and it would be better if such tools can be embedded into GAC.
  Various tools are available for extracting TCGA data (now available through GDC data portal). Data can be downloaded directly from the GDC data portal ( https://gdc-portal.nci.nih.gov/) or by use of R packages.   Some of the more recent packages include TCGA Biolinks (Colaprico *et. al*, 2015) which is available through Bioconductor and the TCGA Assembler (Zhu *et. al*, 2014) available as an R package. Users may refer to the detailed tutorials of these tools for instructions on extracting TCGA data types.   
2. The authors stated that ‘superpc' is unable to address the limitation about clinical association based on a binary outcome in the Introduction. But in the Methods section, it said ‘SuperPC for binary outcome follows the same analysis workflow as the other two outcomes’. It confuses me and please explain the details.
  We have included a sentence in the methods section that clarifies how the superPC tool has been extended to include analysis of binary outcome data.  
3. I find that the calculation will be automatically triggered when changes any one option in the sidebar. That is not a user-friendly experience, especially it happens in the ’Super PC Binary Outcome’ module which cannot return the result immediately. Think about that if the users want to change two default options, they have to adjust the first one, wait a few seconds and then adjust the second option, which is not efficient. So adding a ‘submit’ button to trigger the calculation is a better way.
  A ‘Run Analysis’ submit button is now available under each analysis option that will update results based on user-specified options to multiple parameters, as opposed the previous version which triggered a calculation after each separate change.  
4. Build a user manual page to explain the details of each function and use an example to demonstrate the usage. 
  We have included a tutorial as a separate tab within the tool to demonstrate the usage of each function with example data.

 

**References:**

Colaprico A, Silva TC, Olsen C, Garofano L, Cava C, Garolini D, Sabedot T, Malta TM, Pagnotta SM, Castiglioni I, Ceccarelli M, Bontempi G and Noushmehr H (2015). “TCGAbiolinks: An R/Bioconductor package for integrative analysis of TCGA data.” *Nucleic Acids Research*. doi: 10.1093/nar/gkv1507, http://doi.org/10.1093/nar/gkv1507.

Genomic Data Commons (GDC) Data Portal: https://gdc-portal.nci.nih.gov/. NIH and NCI, accessed in August 2016.

Y. Zhu, P. Qiu, Y. Ji. TCGA-Assembler: Open-Source Software for Retrieving and Processing TCGA Data. Nature Methods. Vol. 11, No. 6, pp:599-600, 2014. | doi:10.1038/nmeth.2956
